# Effect of subsequent bladder cancer on survival in upper tract urothelial carcinoma patients post-radical nephroureterectomy: a systematic review and meta-analysis

**DOI:** 10.1186/s12894-023-01387-3

**Published:** 2023-12-21

**Authors:** Haopu Hu, Shicong Lai, Mingrui Wang, Xinwei Tang, Chin-hui Lai, Kexin Xu, Tao Xu, Hao Hu

**Affiliations:** Department of Urology, Peking University People’s Hospital, Beijing, China

**Keywords:** Cancer- specific survival, Upper urinary tract urothelial carcinoma, Subsequent bladder cancer, Overall survival

## Abstract

**Background:**

Radical nephroureterectomy (RNU) is the primary treatment strategy for upper tract urothelial carcinoma (UTUC). However, the intravesical recurrence occurs in 20–50% of all patients. The specific effect of subsequent bladder cancer (SBCa) on survival remains unclear. Therefore, we investigated the effect of SBCa following RNU in patients with UTUC.

**Methods:**

PubMed, EMBASE, and Cochrane Library were exhaustively searched for studies comparing oncological outcomes between SBCa and without SBCa. Standard cumulative analyses using hazard ratios (HR) with 95% confidence intervals (CI) were performed using Review Manager (version 5.3).

**Results:**

Five studies involving 2057 patients were selected according to the predefined eligibility criteria. Meta-analysis of cancer-specific survival (CSS) and overall survival (OS) revealed no significant differences between the SBCa and non-SBCa groups. However, subgroup analysis of pT0-3N0M0 patients suggested that people with SBCa had worse CSS (HR = 5.13, 95%CI 2.39–10.98, *p* < 0.0001) and OS (HR = 4.00, 95%CI 2.19–7.31, *p* < 0.00001).

**Conclusions:**

SBCa appears to be associated with worse OS in patients with early stage UTUC. However, caution must be taken before recommendations are made because this interpretation is based on very few clinical studies and a small sample size. Research sharing more detailed surgical site descriptions, as well as enhanced outcome data collection and improved reporting, is required to further investigate these nuances.

## Highlights

- RNU is the standard treatment for UTUC.
- SBCa after RNU is common.
- In patients with UTUC, SBCa has no significant effect on prognosis.
- In patients with early stage UTUC, SBCa suggests a worse prognosis.

## Introduction

Upper urinary tract urothelial carcinoma (UTUC) is a rare malignant disease that accounts for 5–10% of all urothelial carcinoma [1]. Radical nephroureterectomy (RNU) with ipsilateral bladder cuff excision (BCE) remains the standard treatment for nonmetastatic UTUC [1, 2]. Patients with early stage tumors generally have a reported 5-year estimated cancer- specific survival (CSS) of > 90%, whereas the 5-year CSS rate in patients with advanced stage tumors is usually less than 50% [3]. Although the pathological T and N categories are stable indicators of UTUC prognosis, it remains necessary to identify more prognostic factors that may guide patient counselling, follow-up scheduling, and the administration of adjuvant therapies [3, 4].

Developing subsequent bladder cancer (SBCa) following radical surgery is common, occurring in between 20 and 50% of all UTUC patients [1, 5], and most of them occur within 1 year postoperatively. The precise etiology of secondary bladder cancer remains ambiguous. The field-cancerization hypothesis suggests that in the background of upper urinary tract urothelial carcinoma (UUC), the entire urothelial lining of the upper urinary tract is exposed to carcinogenic injuries, resulting in the multifocal occurrence of malignant lesions [6]. On the other hand, the tumor seeding theory, posits that cancer cells shed from existing tumors can disseminate and implant in other areas, giving rise to secondary tumors. The multifocal nature of the lesions observed in cases of upper tract urothelial carcinoma (UTUC) often poses a challenge in treatment, thus necessitating a comprehensive approach to manage both the primary tumor and potential secondary lesions [6].

According to the European Association of Urology (EUA) Guidelines, cystoscopy should be performed every 3 months for 1 year after surgery and then at increasing intervals in patients with epithelial carcinoma of the upper urinary tract [1]. Hence, regular bladder examinations should be performed during treatment of patients with UTUC. Although regular cystoscopic review plays an important role in the management of patients with UTUC, as an invasive procedure, the associated bleeding, injury, and cost cannot be ignored [7].

To customize postoperative management of UTUC patients, one current direction of focus is the stratification of patients’ risk of intravesical recurrence. Numerous studies have been designed to identify potential SBCa predictors after RNU [8–10]. However, little is known about how SBCa affects prognosis, and current evidence is sparse and remains controversial. For example, Lee et al. conducted a study which suggested that SBCa is not correlated with long-term oncological outcomes [11]. Likewise, Elalouf et al. found that overall SBCa does not affect prognosis, but they also found that muscle-invasive bladder cancer (MIBC), as opposed to superficial BCa, was associated with worse cancer-specific survival [12]. Conversely, Jiang et al. found that organ-confined UTUC patients who developed SBCa after RNU had significantly higher cancer-specific mortality rates [13]. We believe that while identifying which patients with UTUC are more likely to relapse, attention should also be paid to the specific impact of SBCa on survival. Identifying which patients with SBCa are more likely to affect prognosis can also provide guidance for the postoperative management of UTUC patients.

As such, we sought to systematically review and meta-analyze the best available evidence on the effect of SBCa on prognosis. The overarching aim is to develop and share a knowledge base to support screening and treatment guideline development.

## Methods

### Search and selection

Two authors (SC-L and HP-H) performed a systematic search of PubMed, EMBASE, and Cochrane Library from inception until August 20, 2021. The search strategy involved only clinical studies assessing the oncologic impact of SBCa following radical surgery in patients with UTUC.

The search terms were upper urinary tract urothelial carcinoma, transitional cell carcinoma of the upper urinary tract, upper tract urothelial cancer, upper tract urothelial neoplasms, and radical nephroureterectomy. Additional manual searches were conducted for pertinent studies and citations. The search strategy was designed according to the Preferred Reporting Items for Systematic Reviews and Meta-analysis (PRISMA) statement and AMSTAR (Assessing the Methodological Quality of Systematic Reviews) Guidelines [14].

### Inclusion criteria

Studies were included, if they met all of the following criteria:

1. Patients with primary UTUC without distant metastases or bilateral synchronous upper urinary tract tumors at the time of diagnosis
2. All patients underwent RNU with bladder cuff excision (BCE).
3. The median follow-up period was > 12 months.
4. Oncologic outcomes included cancer-specific survival (CSS) and overall survival (OS).
5. Survival data included hazard ratios (HR) and corresponding 95% confidence intervals (CI) or Kaplan–Meier curves comparing survival between SBCa and without SBCa.
6. Prospective or retrospective studies analyzing the relationship between SBCa and UTUC prognosis.

When multiple studies reported findings based on an identical study population, only the study with the most detailed information was included in the analysis.

### Systematic review process

**Fig. 1:**
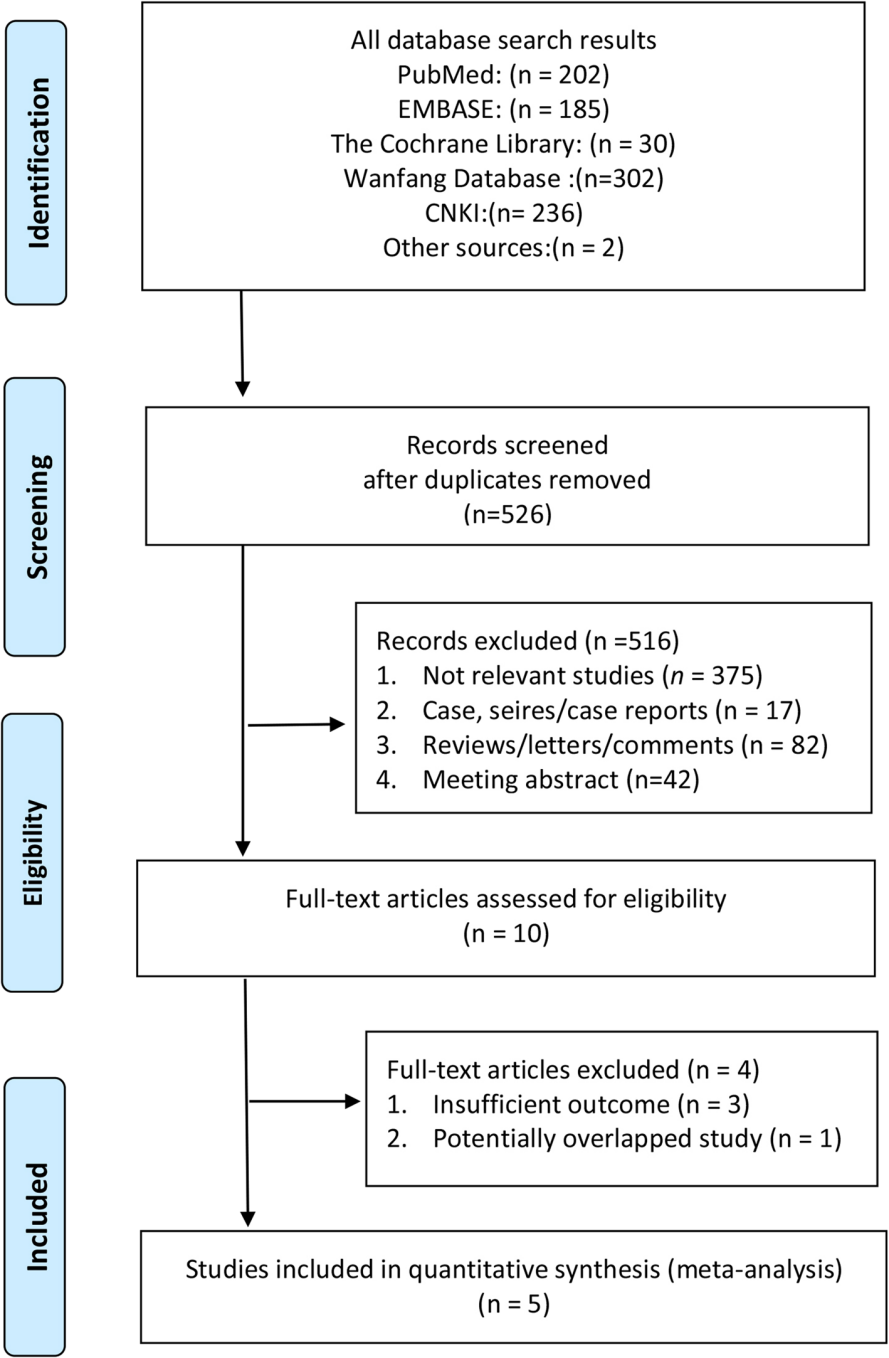
Flowchart of study selection

After duplicates were removed, two authors (HP-H and SC-L) independently reviewed 957 reports. Discrepancies were resolved through consensus. Eventually, only five studies were selected for data extraction and quality assessment. A PRISMA flowchart depicting the review process is presented in Fig. 1.

### Data extraction

Data were independently extracted from full-length articles by two reviewers (HP-H and SC-L) using a standardized item form. Extracted information included author/s, year of publication, country/region, type of study, sample size, number of participants in each group, mean/median age, sex, tumor location and grade, pathological stage, perioperative chemotherapy, median follow-up, and outcomes, including CSS and OS.

### Quality of data assessment

The quality of studies was assessed independently by two reviewers (HP-H and SC-L) using the Newcastle–Ottawa scale (NOS), which is recommended for the assessment of non-randomized studies [15]. The NOS assesses risk across three domains: patient selection, comparability of groups, and outcomes. Studies with NOS scores ≥6 were considered eligible for inclusion in the meta-analysis. Any divergence of opinion was settled through discussion or arbitration by a third reviewer.

### Statistical analyses

Log HR and variance were extracted from all studies and synthesized. For each trial, HRs for survival with corresponding 95% CIs were analyzed in terms of the impact of SBCa following RNU on oncologic outcomes. Given that this evidence base is relatively new, we adopted the assumption that the effects would vary; therefore, the random-effects model (DerSimonian and Laird method) was applied. This model generates more conservative estimates [16].

Sensitivity analysis was performed if there were high levels of heterogeneity. This study aimed to assess the reliability of the findings and identify potential sources of heterogeneity. Subgroup analysis was performed for factors (T, N stage) that clearly influenced prognosis.

Publication bias was assessed by using funnel plots. All data analyses were performed using Review Manager (version 5.3; The Nordic Cochrane Centre, Copenhagen, Denmark). Statistical significance was set than 0.05.

## Results

The search and selection strategy yielded six publications consisting of five separate clinical studies [11–13, 17–19]. Two reports were based on the same trial conducted over different periods [12, 19]. Finally, 2057 participants were included, of whom 138 had been diagnosed with SBCa. A summary of demographics, study design, and clinical characteristics for each of the included studies is provided in Table 1.

**Table 1:** Study characteristics, basic demographics and clinical characteristics

Author/s (year)	Study design	Country	Number of patients		Age (years)	Sex (M/F)	Tumor stage	Lymph node stage	Tumor grade	Follow-up (months)	Outcome/s	NOS
			SBCa	Non-SBCa								
Jiang et al. (2020)	Retrospective,Single center	China	42	187	66.7 ± 9.8	M (117) F (112)	Ta-1 (78) T2 (114) T3 (37)	N0 (229)	Low (37) High (192)	40 (IQR24,56)	CSS OS	8
Elalouf et al. (2013)	Retrospective,Single center	French	85	152	68.5 ± 10.4	M (161) F (76)	T1 (139) T2 (22) T3–4 (76)	N0 (67) Nx (147) N+ (23)	Low (43) High (194)	44 (IQR24,73)	CSS	7
Yamashita et al. (2016)	Retrospective,Multi center	Japan	205	329	72 (30–91)	M (363) F (171)	Ta-1 (219) T2–4 (315)	N0 (244) Nx (239) N+ (51)	Low (26) Intermediate (218) High (273) Unknown (48)	47 (1–78)	CSS OS	7
Lee et al. (2017)	Retrospective,Multi center	Korea	231	529	65.6 ± 10.0; 65.2 ± 9.5	M (561) F (199)	Ta (64) T1 (264) T2 (127) T3 (296) T4 (9)	N0 (26) Nx (711) N+ (23)	Low (229) High (531)	45 (IQR3,76)	CSS OS	7
Elawdy et al. (2017)	Retrospective,Single center	Egypt	139	158	59.0 ± 11.0	M (262) F (35)	T1 (194) T2 (43) T3 (59) T4 (1)	Nx(297)	Low (13) Intermediate (184) High (100)	34 (6–300)	CSS	6

*Abbreviations:* *IQR* Interquartile range, *NOS* Newcastle-Ottowa Scale, *CSS* cancer specific survival, *OS* overall survival

These studies were conducted in Asia (*n* = 3), Africa (*n* = 1), and Europe (*n* = 1). Two multicenter and three single-center studies were conducted. All studies were retrospective, and the largest study was conducted in Korea with 760 participants. Their ages ranged from to 30–91 years, as seen in the Yamashita et al. study in Japan, but generally appears to be around 68 years (± 10 years). Approximately 71% of the participants in this study were men. 46.6% (*n* = 958) were at pTa-1 stage with the remainder having muscle-invasive tumors (pT2–4). 46.6% (*n* = 97) also had lymph node positive tumors.

Overall, the median follow-up period for this sample ranged from 19 to 57.5 months. No significant differences were observed within this sample. After assessment, each study was found to have level III evidence, with scores of ≥6. This evidence is broadly considered adequate for meta-analysis.

### Impact of SBCa on CSS

#### Overall

**Fig. 2:**
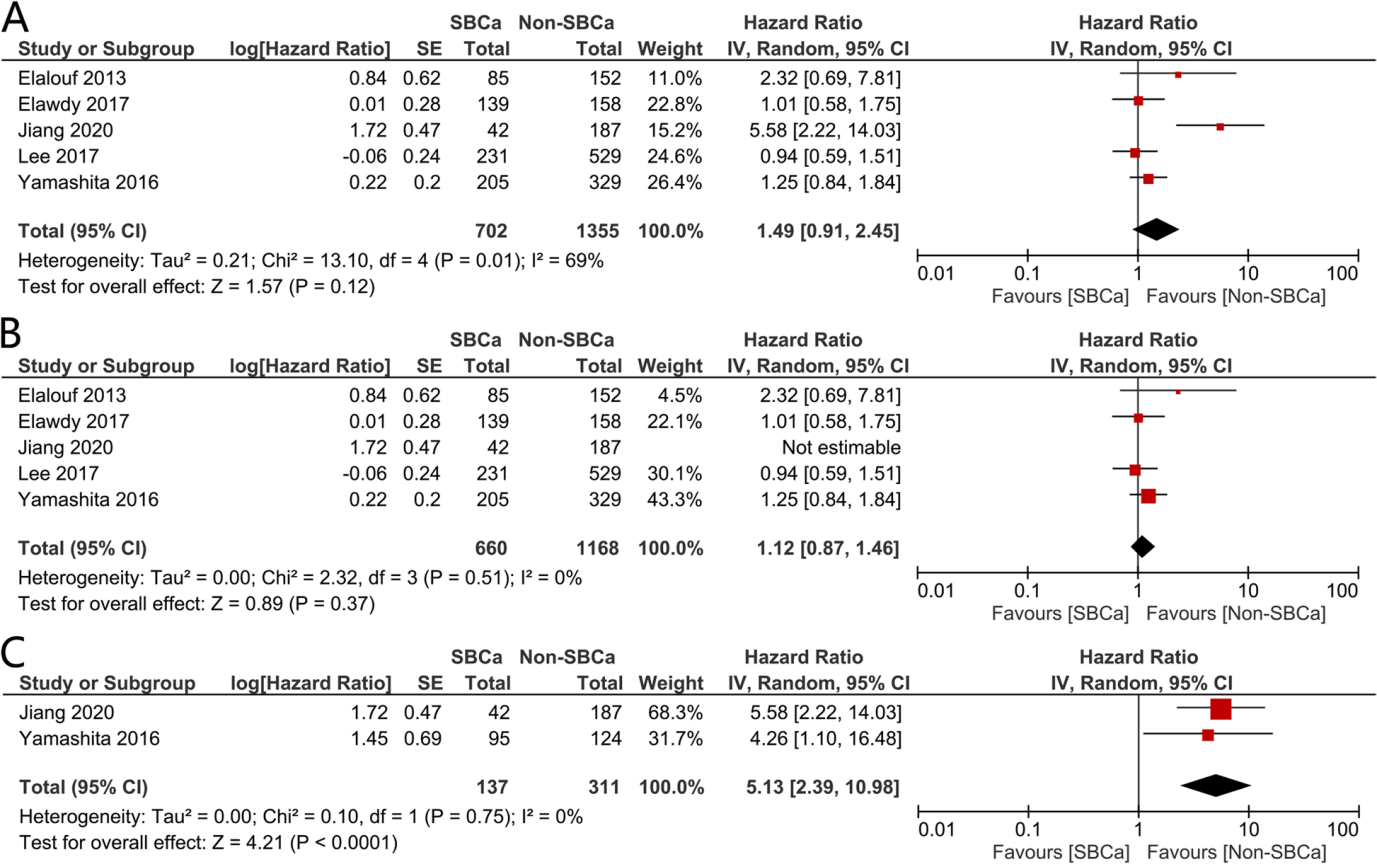
Forest plots assessing the impact of SBCa on css: (**A**) overall population, (**B**) sensitive analysis, (**C**) sub-group analysis

Pooled analysis suggested that there was no significant difference between the two groups (HR = 1.49, 95% CI: 0.91–2.45, *p* = 0.12, Fig. 2A). Pooled analysis revealed that there was significant heterogeneity between the trials (I2 = 69%). Therefore, sensitivity analysis was then performed to reduce the heterogeneity and confirm the result.

#### Sensitivity analysis

**Fig. 3:**
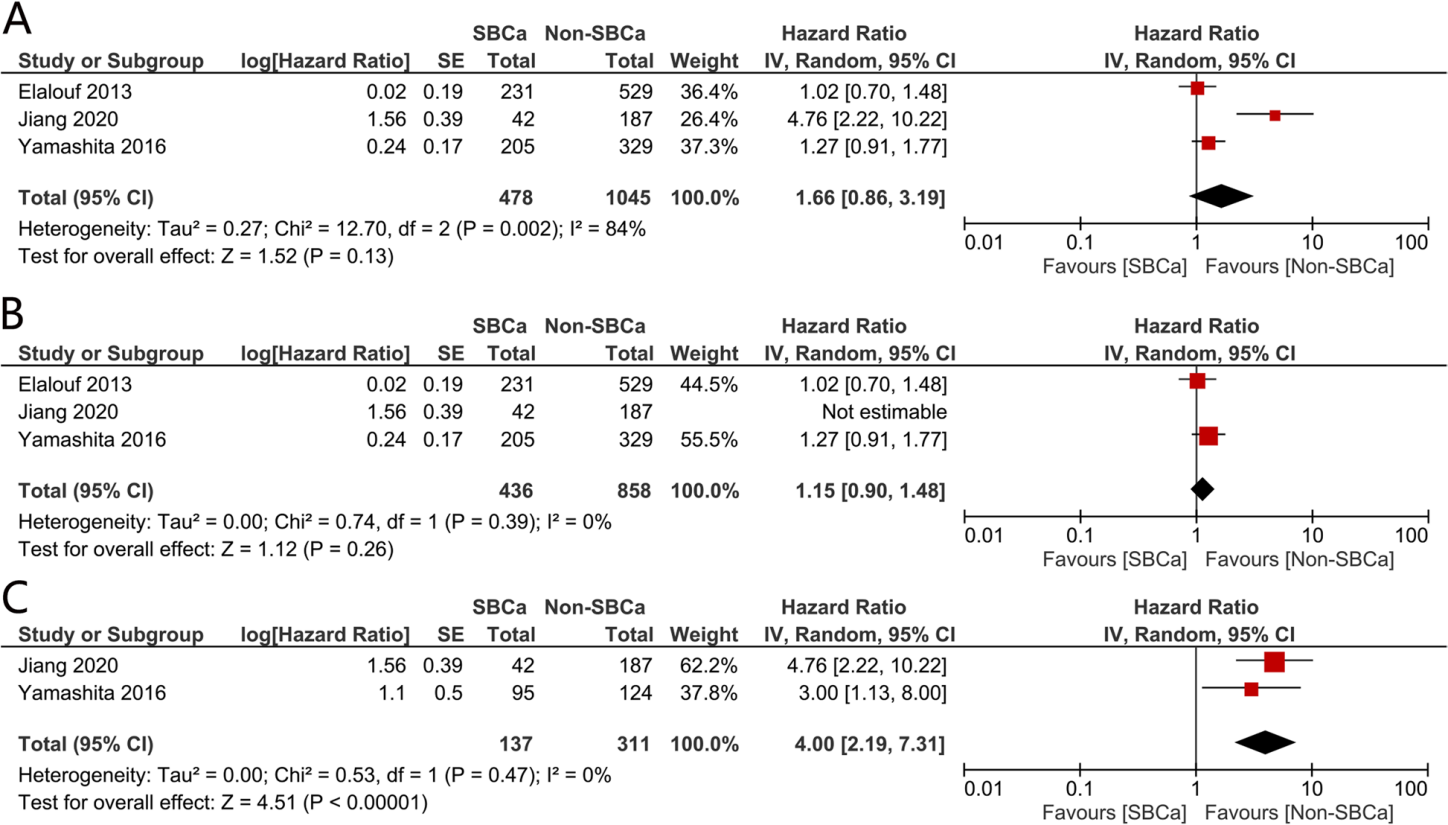
Forest plots assessing the impact of SBCa on os: (**A**) overall population, (**B**) sensitive analysis, (**C**) sub-group analysis

A sensitivity analysis was performed by removing trials that included only T3N0M0 patients (Fig. 3c). This technique lowered the level of heterogeneity substantially to I2 = 0% and highlighting the source of heterogeneity. The recalculated results then suggested that the association between SBCa and CSS was not statistically significant (HR = 1.12, 95% CI: 0.87–1.46, *p* = 0.37, Fig. 2B).

#### Subgroup analysis

Since pathological T and N stages had a significant influence on survival, further subgroup analysis excluding T4 or node-positive patients was conducted. Finally, two trials involving 448 patients were included, and the combined HRs revealed that patients with SBCa had significantly worse survival (HR = 5.13, 95% CI: 2.39–10.98, *p* < 0.0001, Fig. 2C).

### Impact of SBCa on OS

#### Overall

Three studies involving 1523 patients reported the OS and were included in this meta-analysis. Results suggested that there was no significant difference between the SBCa and non-SBCa groups (HR = 1.66, 95% CI: 0.86–3.19, *p* = 0.13, Fig. 3A). However, significant heterogeneity was detected between the trials (I2 = 84%), which initiated sensitivity analysis.

#### Sensitivity analysis

Sensitivity analysis was performed by removing trials that only included T3N0M0 patients (Fig. 3c), which lowered the level of heterogeneity to I2 = 0%. Recalculated results however also indicated that SBCa was not significantly related to OS (HR = 1.15, 95% CI: 0.90–1.48, *p* = 0.26. Please refer to Fig. 3B for further details.

#### Subgroup analysis

Subgroup analysis involved the exclusion of T4 or node-positive patients. OS data were reported in only three studies, and pooled analysis suggested that patients with SBCa do have significantly poorer survival (HR = 4.00, 95% CI: 2.19–7.31, *p* < 0.00001, Fig. 3C). There was No apparent heterogeneity (I2 = 0%, *p* = 0.47), in this instance.

### Publication bias

**Fig. 4:**
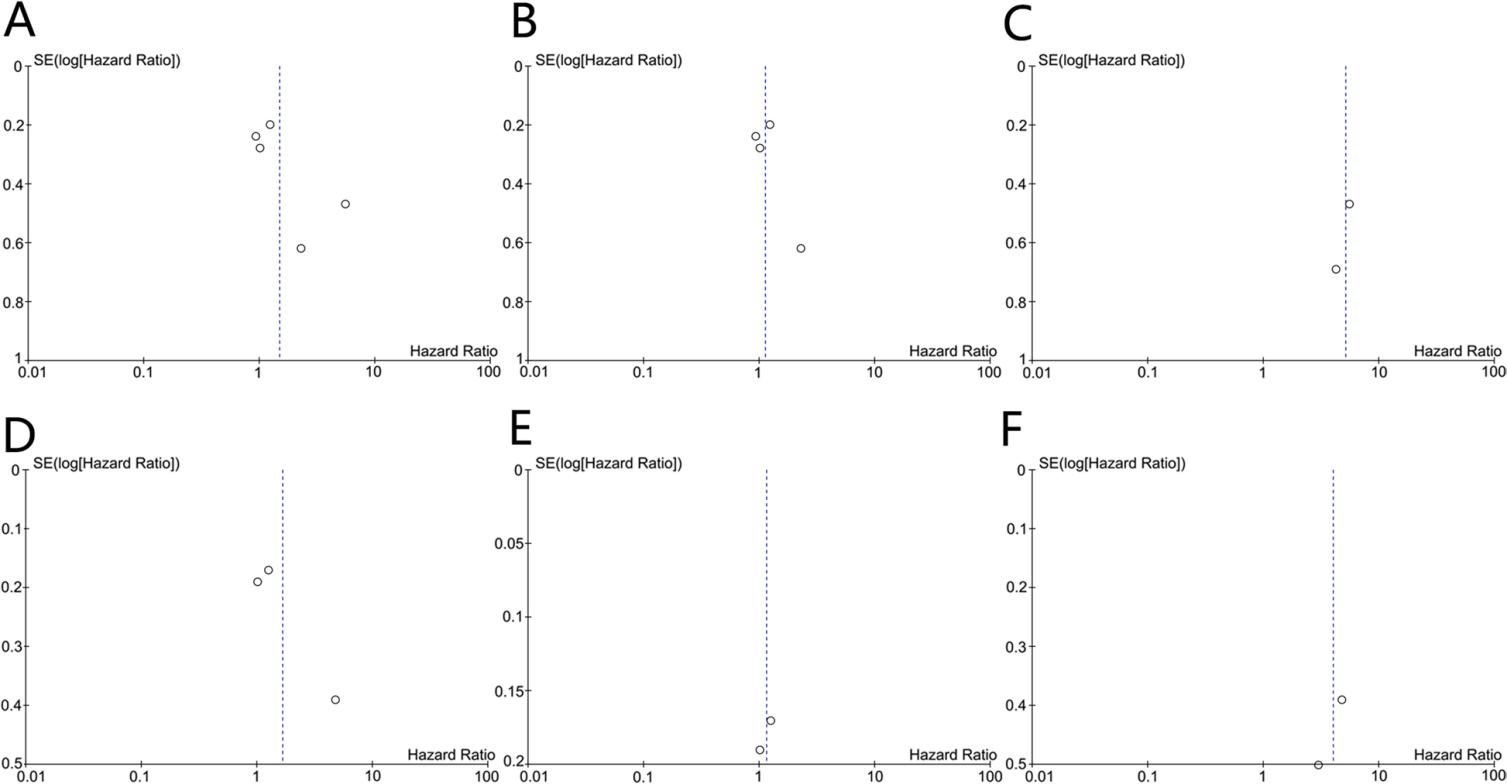
Funnel plot assessing publication bias

The basic symmetry of the funnel plots suggested that there was no obvious publication bias across this evidence base at this stage (Fig. 4).

## Discussion

Systematic search and strict eligibility criteria ensured that we could synthesize evidence from five studies involving 2057 people. Participants were predominantly men (≈71%) and were aged between 30 and 91 years. All the included studies were retrospective, with follow-up periods ranging from 1 to 300 months. This sample of studies can be considered to have a reasonably high level of evidence, despite the relatively small number of studies. The meta-analysis suggested that there was no significant difference between those who developed SBCa and those who did not. However, subgroup analysis of pT0-3N0M0 patients appears to confirm that people who develop SBCa are at a greater risk of lower cancer-specific survival and overall survival.

For localised or locally advanced UTUC, RNU with BCE provides lasting local control but CSS rates can be as hgh as 70% [20, 21]. However, the recurrence and progression rates of this type of malignant tumor remain high [1, 5]. Like most other malignancies, pathological stage is the strongest predictor of survival in patients with UTUC [3, 20, 22]. However, its predictive value as an individual risk factor has been limited. Given the relative rarity of this disease, analyses such as this are unlikely to identify stronger predictive values. As such, there is an increasing awareness of data mining, specifically in oncology, which will enable us to identify further risk factors affecting survival, although at present, we do not have the breadth of data required. Thus, even though there are a few studies available in this field, little is currently known about SBCa rates following radical surgery, how patients respond, prognosis, and the importance of timing treatments such as chemotherapeutics.

In this study, meta-analytical techniques were adopted to synthesize evidence, not only to produce more reliable recommendations, but also to identify issues that require further consideration in primary research. First, we analyzed the differences in CSS and OS between the SBCa and non-SBCa groups. These results are consistent with the individual studies of Lee et al., Elalouf et al., and Elawdy et al. [12, 18]. We found that SBCa after radical surgery had no significant effect on CSS and OS; however, each individual study had limitations that may have affected this analysis. For example, most of the included studies only provided single-factor analysis; therefore, we could not obtain the data after the correction of other factors, such as tumor stage, grade, age, and sex. Furthermore, Elawdy et al. showed a comparatively lower mean age of participants, while the median duration of follow-up was relatively shorter. The study conducted by Jiang et al. encompassed a larger proportion of individuals with muscular infiltrating UTUC within the sampled population. This was likely to have caused a degree of bias in the obtained effect values.

To reduce the amount of bias related to tumor staging in this meta-analysis, we performed a subgroup analysis that excluded T4 or node-positive patients. These findings suggest that patients with SBCa have significantly poor survival rates. Again, this finding is in keeping with previous reports in which researchers observed that SBCa is a predictor of both overall survival and cancer specific survival in the organ-confined UTUC population [13]. Non-muscle-invasive UTUC has also been confirmed as a predictor of poor survival in patients with SBCa [17]. While this appears to be self-evident, it also raises a number of questions regarding seeding and cancerization, which ought to be addressed.

There are two main theories that may account for these findings: the field-cancerization hypothesis and tumor seeding theory [23–25]. Field cancerization is a hypothesis that assumes that multifocal urothelial carcinoma arises secondarily, and perhaps independently, within the urothelial tract as a consequence of potentially external cancer-causing factors such as smoking and the use of specific pharmaceuticals. The tumor seeding theory on the other hand, adopts the rather prosaic analogy of cancer as a flower, budding and then seeding [6, 26, 27]. While both the field cancerization hypothesis and tumor seeding theory have merits, the seeding theory is more likely in this instance because of the urological procedures involved. However, further research is necessary to understand subsequent tumor location and genetic instability.

According to recent studies by Du et al. and Li et al., urothelial carcinoma at different sites can occur with an independent clonal origin. In some instances, subsequent tumors can be both polyclonal and monoclonal, which raises another set of questions regarding the mechanisms involved in SBCa [24, 28]. Indeed, many academics and clinicians believe that SBCa, which occurs during the early postoperative period, can be attributed to the spread and planting of primary carcinoma cells, although this may be related to soiling and perhaps urothelial injury during surgery, which may encourage cellular adherence [17, 29, 30]. This issue requires further attention. However, at present, we do not have the data, nor are we designing studies that incorporate a more complex set of indicators for analysis. This should not be entirely left to basic medical researchers. With more rigorous clinical study designs, we can gain insights into the mechanisms involved.

However, SBCa research has revealed that tumor cells are commonly and more aggressive after RNU. As we are acutely aware, quicker cell proliferation and growth correspond with worsening prognosis, which means that postoperative surveillance must be enhanced. At present, guidelines and the included studies appear to have adopted standard follow-up procedures, but seeding (and indeed cancerization) may also be related to tumor size and injury or surgical methods, such as open or laparoscopic approaches [1]. At present, there are few indicators and effective interventions. One may advocate indiscriminate adjuvant intravesical instillation with chemotherapy; however, this could have a greater impact on a patient’s physical and emotional well-being.

Therefore, more active postoperative surveillance and a more accurate predictive model for SBCa after RNU should be proposed for patients with UTUC. This may help improve the long-term prospects of these patients. By incorporating the results of this study, we can conduct more frequent cystoscopy and intervention therapy for relatively early stage UTUC patients with a higher risk of SBCa during clinical follow-up. This is because preventing SBCa in this group of patients can provide the greatest survival benefit. For patients with relatively advanced-stage disease, palliative treatment should focus on avoiding late-stage symptoms. To facilitate urologists in designing individualized monitoring strategies based on individual patient risk factors.

Furthermore, in addition to traditional clinical indicators, biomarkers or genetic analyses related to bladder recurrence after UTUC surgery have received extensive attention. For example, the preoperative neutrophil-to-lymphocyte ratio (NLR) has been shown to correlate with tumor invasiveness [31]. Inoue et al. studied the expression of vascular generation- and invasion-related genes in 55 UTUC patients who underwent RNU and found that E-cadherin expression was associated with bladder-specific recurrence [32].

With the application of next-generation sequencing technology, genetic analysis is also significant for predicting SBCa. Forkhead box O3A (FOXO3A), which belongs to the FOXO protein family and is located on human chromosome 6q21, is usually involved in DNA damage repair, cell cycle regulation, apoptosis, and the cell stress response as an important transcriptional regulator. Downregulation of FOXO3A expression may promote the occurrence, metastasis, and progression of UTUC [33]. François et al. used targeted next-generation sequencing to prospectively sequence tumors and found a significant correlation between the risk of bladder recurrence after radical surgery for UTUC and mutations in FGFR3, KDM6A, CCND1, and TP53 [34]. In a multicenter retrospective study by Soria et al., which included 732 UTUC patients after RNU, 35.8% of patients had overexpression of HER2, which was associated with SBCa [35].

From a strictly surgical perspective, radical resection must comply with oncological principles; that is, surgery of this nature must also attempt to prevent tumor seeding by avoiding entry into the urinary tract during surgery [21]. However, previous high-quality evidence has demonstrated that a single postoperative dose of intravesical chemotherapy (pirarubicin and mitomycin C) soon after surgery (< 72 h) reduces the risk of SBCa occurrence within the first year post-RNU [36–39]. At present, this is administered at the physician’s discretion; however, this may need to become a standard procedure to reduce the likelihood of cancerization and seeding.

To the best of our knowledge, this is the first study to assess the effect of SBCa on the survival of patients with UTUC using standard meta-analytical techniques. However, prior to providing recommendations, some limitations should also be considered. As a meta-analysis, this study has inherent methodological limitations that are difficult to avoid. First, owing to data source restrictions, this study relied on aggregated data rather than individual patient data. Second, given the low incidence of the disease, high-quality studies are scarce, which may introduce bias. Additionally, the inclusion of only retrospective studies in this analysis hampers the examination of crucial factors, such as detailed bladder irrigation chemotherapy regimens, which could contribute to our progress. Moreover, there may be a need to incorporate more surgical details, surgeon-specific experience, and skills. There may be an increased risk of seeding through biopsy and surgical resection [40]. Theoretically, circulating cancer cells may be encouraged to leave the upper urinary tract, disseminate to distant sites, and inadvertently seed at the sites of surgical injury. Therefore, future prospective studies on patients with UTUC should encompass more intricate details, including surgical expertise, surgical details, biomarkers, and genetic analyses.

## Conclusions

In early stage UTUC populations, SBCa appears to be associated with worse survival; however, caution must be exercised because this finding is based on a very limited number of clinical studies. Research sharing more detailed surgical site descriptions, as well as enhanced outcome data collection and improved reporting, is required to investigate the nuances involved.

## Data Availability

The datasets generated or analyzed during this study are available from the corresponding author upon reasonable request.

## Abbreviations

AMSTAR: Assessing the methodological quality of systematic reviews
BCE: Bladder cuff excision
CI: confidence interval
CSS: Cancer-specific survival
HR: Hazard ratios
NOS: Newcastle–Ottawa scale
OS: Overall survival
PRISMA: Preferred Reporting Items for Systematic Reviews and Meta-analysis
RNU: Radical nephroureterectomy
SBCa: Subsequent bladder cancer
UTUC: Upper tract urothelial carcinoma
